# Dissipation Kinetics and the Pre-Harvest Residue Limits of Acetamiprid and Chlorantraniliprole in Kimchi Cabbage Using Ultra-Performance Liquid Chromatography-Tandem Mass Spectrometry

**DOI:** 10.3390/molecules24142616

**Published:** 2019-07-18

**Authors:** Jonghwa Lee, Byung Joon Kim, Eunhye Kim, Jeong-Han Kim

**Affiliations:** 1 Department of Agricultural Biotechnology and Research Institute of Agriculture and Life Sciences, Seoul National University, Seoul 08826, Korea; 2 Department of Veterinary and Animal Sciences, University of Massachusetts, Amherst, MA 01003, USA

**Keywords:** pesticide residue, pre-harvest residue limit, acetamiprid, chlorantraniliprole, kimchi cabbage

## Abstract

The dissipation behaviors of acetamiprid and chlorantraniliprole in kimchi cabbages were studied under open-field conditions. A simple and rapid analytical method was developed using ultra-high performance liquid chromatography coupled with tandem mass spectrometry (UHPLC-MS/MS). The multiple reaction monitoring (MRM) conditions of two pesticides were optimized to quantify and identify the pesticide residues. Sample preparation was performed by the QuEChERS (quick, easy, cheap, effective, rugged, and safe) method. Average recovery rates at the different spiked levels (0.05 and 0.25 mg/kg) were in the range of 103.6–113.9% (acetamiprid) and 80.8–91.2% (chlorantraniliprole), and the relative standard deviations were ≤4.3% for all. The dissipation kinetics were assessed using first-order equations after spraying acetamiprid and chlorantraniliprole individually on kimchi cabbages. The biological half-lives in field 1 and 2 were 5.2 and 6.3 days (acetamiprid) and 10.0 and 15.2 days (chlorantraniliprole), respectively. Based on the dissipation equations, the pre-harvest residue limits (PHRLs) corresponding to each day before harvest were suggested as the guidelines to meet the MRL on harvest day. It was also predicted that the terminal residues observed after multiple sprayings (three and seven days) would be below the MRL when harvested, in compliance with the established pre-harvest intervals.

## 1. Introduction

Pesticides are chemical substances that are widely used to protect crops against various pests and diseases. The use of pesticides during cultivation has improved the quality of agricultural products and enabled economic farming [1,2]. Although pesticides are indispensable in modern agricultural practices, it is obvious that excessive persistence of pesticide residues is a potential risk to public health and the environment [3,4]. Governments and international organizations are regulating the use of pesticides and setting the acceptable maximum residue limit (MRL) or tolerance to ensure that food is safe. From the moment that agricultural products are harvested, the pesticide residue levels are thoroughly monitored by regulatory authorities as to whether they meet the MRL. Over-limit agricultural products are mostly discarded before they are consumed, which causes both significant economic losses and unintentional costs for disposal. To prevent such losses, a pre-harvest residue limit (PHRL) has been set to assess the safety of pesticide residues during crop cultivation by the Ministry of Food and Drug Safety in the Republic of Korea. The PHRL is a criterion for determining whether the growing crops would meet the MRL by predicting a residual level at the time of harvest. Appropriate measures are taken for potentially unsafe crops that have exceeded the PHRL before use such as delaying the harvest or being altered for another use. Moreover, studying the dissipation behavior and the biological half-life of pesticide residues during cultivation is essential to predicting the PHRLs. A number of published papers have studied the PHRL in various crops and pesticides [5,6,7,8,9].

Kimchi cabbage (*Brassica rapa*) is a member of the genus *Brassica* belonging to leafy vegetables. It has been called various common names such as Chinese cabbage, napa cabbage, or bok choy. The morphological features that distinguish kimchi cabbage from other species are an oblong and tight cylindrical head, semi-enclosed top, and crinkled leaves. In 2013, the name of the kimchi cabbage was officially adopted by the Codex Committee on Pesticide Residues [10]. According to the data from FAO, the production quantity of cabbages and other brassicas was about 2.4 million tonnes, amounting to 73.8% of all domestic vegetables in 2017. Despite the relatively small land area, the Republic of Korea was the fourth largest producer of cabbages following China, India, and Russia [11]. In addition, kimchi cabbage is the primary ingredient of kimchi, which is a staple food in Korea, as well as a major part of the agricultural market. Because of the importance of kimchi cabbage, proper use of pesticides and the control of the residues during kimchi cabbage cultivation have been a major concern for public health.

Among the various pesticides being used during the cultivation of kimchi cabbage, acetamiprid and chlorantraniliprole are widely used insecticides against moths (*Plutella xylostella* and *Spodoptera exigua*), aphids (*Lipaphis erysimi* and *Myzus persicae*), and striped flea beetles (*Phyllotreta striolata*) [12,13]. The neonicotinoid acetamiprid ((E)-*N*^1^-[(6-chloro-3-pyridyl)methyl]-*N*^2^-cyano-N^1^-methylacetamidine) is a systemic insecticide, having a translaminar mode of action in the stomach and acting as an acetylcholine receptor agonist. It is highly water soluble, and the logP K_ow_ is relatively low (0.80 at 25 °C) [13,14]. Chlorantraniliprole (3-bromo-*N*-[4-chloro-2-methyl-6-(methylcarbamoyl)phenyl]-1-(3-chloropyridin-2-yl)-1*H*-pyrazole-5-carboxamide) is a systemic insecticide, belonging to the diamide class of chemistry. Studies on the mode of action of chlorantraniliprole suggest that it generally causes lethargy and muscle paralysis, leading ultimately to the death of the insect. Chlorantraniliprole is known to have low solubility in water (0.9−1.0 mg/L), but is readily soluble in organic solvents with a high K_ow_ logP (2.76 at pH 7) [13]. MRLs for both acetamiprid and chlorantraniliprole have been established for more than a hundred commodities by the Ministry of Food and Drug Safety in Korea. The value of MRL in kimchi cabbage is 1.0 mg/kg for both pesticides [15].

A number of dissipation studies have investigated acetamiprid residues in zucchini, watermelon, chili, tea, asparagus, parsley, and swiss chard [16,17,18,19,20,21,22]. The dissipation of chlorantraniliprole has also been studied in tomato, grape, strawberry, and cauliflower [23,24,25,26]. Chen, et al. [12] performed a similar field study of acetamiprid on Chinese cabbage (*Brassica pekinensis Skeels*). They compared the residue data with the MRL and suggested the pre-harvest interval, but no degradation kinetics or biological half-lives were studied. The dissipation kinetics of acetamiprid have been studied by Fujita et al. [27], but it was performed on cabbage *(Brassica oleracea*), which is a taxonomically different commodity. Kar et al. [28] have also conducted a field trial to determine the chlorantraniliprole residues in cabbage, but the dissipation study was not performed. To the best our knowledge, there is no previous study on the dissipation kinetics of acetamiprid and chlorantraniliprole residues in kimchi cabbage.

The overall aims of this study were: (1) To investigate the dissipation pattern of acetamiprid and chlorantraniliprole by degradation kinetics during the cultivation of kimchi cabbage, (2) to propose a pre-harvest residue limits during cultivation based on the biological half-lives, and (3) to estimate the final residue level based on the directions for using the pesticides.

## 2. Results and Discussion

### 2.1. Optimization of the Instrumental Condition

**Figure 1:**
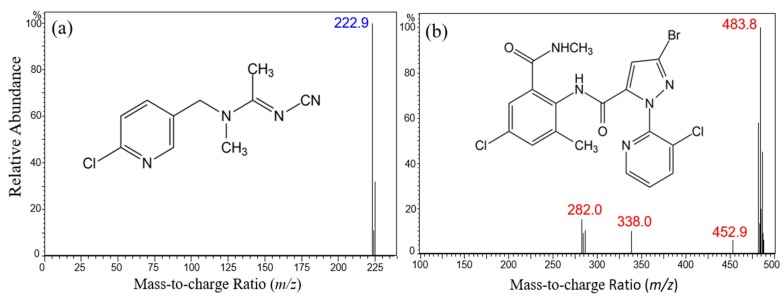
Chemical structures and the mass spectrometry (MS) full-scan spectrum of acetamiprid (**a**) and chlorantraniliprole (**b**).

The multiple reaction monitoring (MRM) optimization of target compounds was performed by injecting individual standard solutions without an analytical column. The full scan spectrums were preferentially obtained to choose an optimal precursor ion. The chemical structures of these two pesticides and obtained spectrums are shown in Figure 1. The protonated molecular ions ([M + H]^+^) of *m/z* 223 (acetamiprid) and *m/z* 484 (chlorantraniliprole) were predominantly observed in positive ionization mode. Since acetamiprid contains one chlorine atom in its molecular structure, the protonated isotopic molecular ion of 225 ([M + H + 2]^+^) was also found at the relative ratio of 3:1 with [M + H]^+^. Many isotopic ions of chlorantraniliprole were produced around the [M + H]^+^ ion, which is caused by the presence of one bromine and two chlorine ions in the molecular structure. The deprotonated molecular ions ([M − H]^−^) were also detected in the negative ionization mode for both compounds, but their intensities were significantly lower than those of the positive ionization mode.

**Figure 2:**
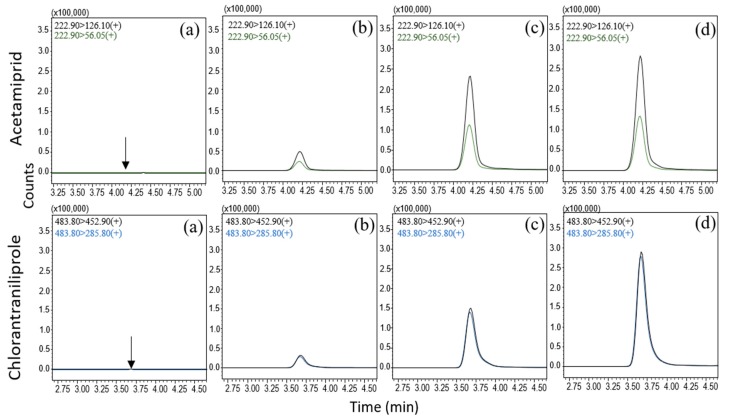
The representative chromatograms of acetamiprid (top) and chlorantraniliprole (bottom): Blank kimchi cabbage (**a**), spiked at 0.05 mg/kg (**b**), spiked at 0.25 mg/kg (**c**), and the field samples two days after spraying (**d**).

After choosing these most abundant ions as the precursor ion, product ion scanning was performed in the various CE ranges (0–50 eV) to identify the product ion that gave the best intensity for the MRM transitions. The optimized MRM transitions, including acquisition parameters, are shown in Table 1. The chromatographic separation was achieved using a short gradient program (12 min for acetamiprid and 7 min for chlorantraniliprole) on a reverse phase C18 analytical column. As shown in the representative chromatograms (Figure 2), no interference was observed in the same retention times of the target compounds, which confirmed that the analytical condition was enough to quantify and identify the pesticide residues.

### 2.2. Method Validation

The multiple reaction monitoring (MRM) optimization of target compounds was performed by injecting individual standard solutions without an analytical column. The full scan spectrums were preferential. Based on the published literature and our experience, both pesticides are known to be well recovered by the QuEChERS (quick, easy, cheap, effective, rugged and safe) method from various commodities like cabbage, fruits, and grains [29,30]. Among the various QuEChERS versions, the citrate-buffered QuEChERS method, which uses citrates for extraction under buffered conditions, was employed for the extraction of acetamiprid and chlorantraniliprole in kimchi cabbage. The accuracy and precision of the analytical method were evaluated by performing three replicates of recovery tests at two spiked levels (0.05 and 0.25 mg/kg). The results in Table 2 show satisfactory recovery rates and relative standard deviations (RSD) in both compounds for all spiked levels. The mean recoveries of acetamiprid in 0.05 and 0.25 mg/kg levels were 103.6% and 113.9% with RSD values of 4.3% and 1.8%, respectively, while chlorantraniliprole had relatively lower recovery rates (80.8% and 91.2%) with RSD values of 2.8% and 0.7%, respectively.

The limit of quantification (LOQ) was defined as the minimum concentration that gives a sufficient signal-to-noise of 10 in the chromatographic signal. The matrix-matched standards treated with the sample preparation method described above were injected into the ultra-high performance liquid chromatography coupled with tandem mass spectrometry (UHPLC-MS/MS) instrument to compare the signal-to-noise ratios. The LOQ was estimated to be 0.005 μg/mL in both of acetamiprid and chlorantraniliprole. Considering the dilution factor of the sample preparation procedure, the concentration of 0.01 mg/kg in both compounds was calculated as the method limit of quantification (MLOQ), which means the lowest detectable concentration in a sample. Given that the registered MRL is 1.0 mg/kg in kimchi cabbage, the analytical method was considered to be sensitive enough for the residue analysis of target pesticides. The linearity (*r*^2^) of the calibration curve was studied based on the matrix-matched calibrations with a concentration range from 0.005 to 0.5 μg/mL. Good linearity with *r*^2^ values of 0.9998 and 0.9964 was obtained for acetamiprid and chlorantraniliprole, respectively. The equations and regression coefficient are presented in Table 2.

Since the harvested samples were stored for 130 days before the analysis, a storage stability test was conducted to confirm that the pesticide residues were not degraded under the same storage condition. To verify the stability of the target pesticide during frozen storage (−20 °C), the stability test was carried out using the spiked samples. Samples spiked at a concentration of 0.25 mg/kg (*n* = 3) were stored in the same freezer as the field samples and were also analyzed on the same day as the field samples. The mean recovery rates of the stability test were 114.1% (acetamiprid) and 96.7% (chlorantraniliprole) with an acceptable RSD value. These results indicated that both pesticides were stable in frozen samples up to 130 days. As a result, the above findings demonstrated that the analytical method was reliable for the quantification of acetamiprid and chlorantraniliprole residues for the field sample.

### 2.3. Dissipation Patterns and Biological Half-Lives of Acetamiprid and Chlorantraniliprole

**Figure 3:**
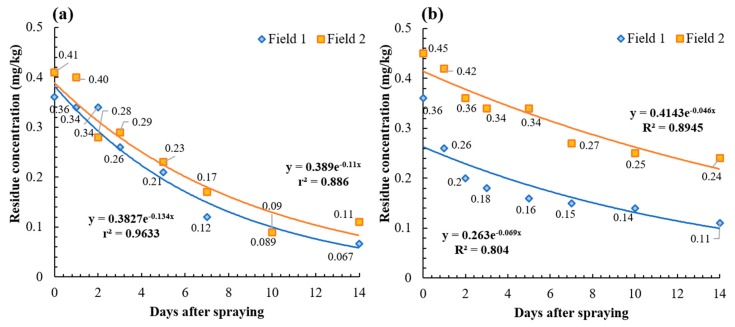
Dissipation patterns of acetamiprid (**a**) and chlorantraniliprole (**b**) in kimchi cabbage in fields 1 and 2.

The dissipation characteristics were determined by measuring pesticide residues of acetamiprid and chlorantraniliprole from the field samples (Figure 3). The initial residue amounts (day 0) of acetamiprid and chlorantraniliprole in field 1 and 2 were found to be 0.36–0.41 mg/kg and 0.36–0.45 mg/kg, respectively, which were quite comparable. In all cases, the initial residues did not exceed the MRLs (1.0 mg/kg) set by the Ministry of Food and Drug Safety in the Republic of Korea. The similar initial deposits of two pesticides may be attributed to the similar contents of the active ingredients (5% for acetamiprid and 6% for chlorantraniliprole) and the same dilution factor of pesticide formulation (2000 times dilution) for spraying.

The dissipation equations of acetamiprid by first-order kinetics were C_t_ = 0.3827 *e*^−0.134t^ (field 1) and C_t_ = 0.3890 *e*^−0.110t^ (field 2). The dissipation dynamic equations of chlorantraniliprole were C_t_ = 0.2630 *e*^−0.069t^ (field 1) and C_t_ = 0. 4143 *e*^−0.046t^ (field 2). Unlike the similar initial deposits, the dissipation pattern over time was significantly different between two pesticides. As shown in Figure 3, the initial residues of acetamiprid were degraded to 0.07–0.11 mg/kg over 14 days, and the half-lives in fields 1 and 2 were 5.3 and 6.3 days, respectively. The initial residual amounts of chlorantraniliprole in fields 1 and 2 were 0.11 and 0.24 mg/kg, respectively, with longer half-lives of 10.0 and 15.2 days. In other words, the biological half-lives of acetamiprid were similar for fields 1 and 2, but those of chlorantraniliprole were different in the two fields. In view of this fact, we confirmed that different degradation behaviors could arise from multiple factors such as the physicochemical properties of the pesticide, application technique, and environmental factors [27,28,31].

Factors affecting the degradation behavior of pesticides applied to crops include physicochemical characteristics of a given pesticide (e.g., vapor pressure, water solubility, hydrolysis), characteristics of the crop commodity (e.g., growth rate, ease of penetration, translocation, excretion), microbial activity, and environmental factors (e.g., rainfall, temperature, sunlight, humidity) [27,32,33,34,35,36]. Acetamiprid has been reported to have a short half-lives in studies of zucchini (1.9 days) [16], chili peppers (2.24–4.84 days) [18], watermelon (3.1–3.9 days) [17], okra fruit (2.3 days) [37], green tea shoots (1.82–2.33 days) [38], and mustard plants (1.02 and 1.59 days) [39]. Previous studies have shown that chlorantraniliprole also tends to degrade rapidly with half-lives of 4.9–5.4 days in corn straw [40], 1.25 and 1.36 days in cauliflower [26], 2.7 days in grape [24], 3.3 days in tomato, and 0.93–1.33 days in berseem leaf. Overall, the dissipation tendencies of both pesticides in this study were slightly slower than those of previous studies. Since an increase in the weight of the kimchi cabbage was observed during the test period (Supplementary Materials, Figure S1), a dilution effect due to the plant growth was not expected, but this is one of the primary factors that may reduce the residue concentration [41,42,43]. This assumption is in agreement with those of previous studies, which have reported relatively long half-lives of chlorantraniliprole in maize straw (9.0–10.8 days) [44] and apple (16–17 days) [45]. In addition, it might be supposed that the absence of heavy rainfall (>2 mm) can cause runoff of the pesticide residue, and the relatively low temperature (4.5–11.6 °C) of the field also contributed to the relatively long half-lives of both pesticides in kimchi cabbage (Supplementary Materials, Figure S2) [46,47].

### 2.4. Estimation of the PHRLs

**Figure 4:**
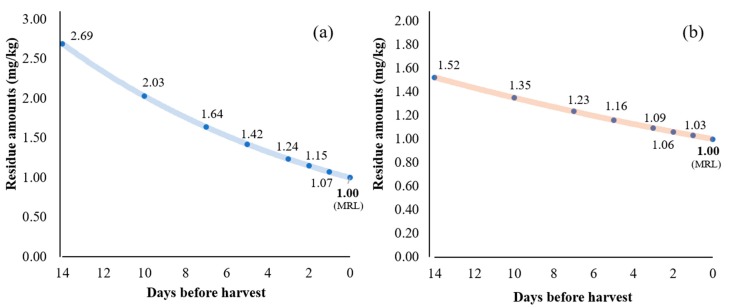
Pre-harvest residue limit curves for acetamiprid (**a**) and chlorantraniliprole (**b**) in kimchi cabbage.

PHRLs of acetamiprid and chlorantraniliprole in kimchi cabbage were calculated based on the pesticide dissipation equations. It was postulated that the pesticide residue amounts of 1.0 mg/kg (which is the MRLs for both pesticides) would be present at harvest. Then, the predicted concentrations from 1 to 14 days prior to harvest were calculated based on the dissipation kinetics equations obtained from field 2, this gave a slow-decline pattern. The lowest value in the confidence interval (Student’s *t*-test at 95% confidence level) of the regression coefficient in the field 2 data was used to predict the PHRLs to assume the worst case scenario. The predicted PHRL curves are presented in Figure 4. It is predicted that if the residual concentration of acetamiprid at 10 days prior to harvest is below 2.03 mg/kg, it is likely that the final residue amount at harvest date will not exceed the MRL. Likewise, the chlorantraniliprole residue concentration of 1.35 mg/kg at 10 days prior to harvest was suggested as a PHRL. This data can be used as a guideline for producing safe agricultural products prior to harvest, thereby ensuring food safety for the consumer.

### 2.5. Prediction of Residue Levels by Multiple Sprayings

Based on the dissipation constants and initial deposits derived from this study, we estimated the residue amounts by possible multiple sprayings that can happen in accordance with the pre-harvest interval (PHI). The PHI is the amount of time (days) between the last application and the harvest, so that the terminal residue declined below the MRL. The PHI of the acetamiprid formulation (6%, SC) was set to seven days after spraying three times, and 14 days after spraying twice for the chlorantraniliprole formulation (5%, WG) in kimchi cabbage. However, there have been no discussions of the application intervals for kimchi cabbage in the directions for use of the pesticide, unlike other crops where 7-day or 10-day intervals have been set. Considering that frequent spraying of pesticides is possible depending on the occurrence of the pest and disease, the terminal residues were simulated after different frequencies of spraying. The dissipation constant and the amount of initial deposit were derived from the data that showed the worst case degradation pattern. It was assumed that the initial residual amount is added to each subsequent application.

**Figure 5:**
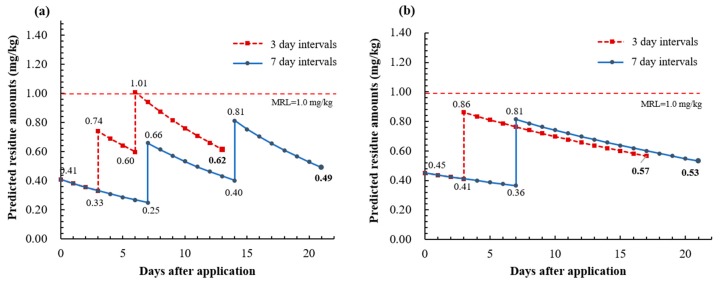
Predicted residue levels for multiple applications (three- and seven-day intervals) of acetamiprid (**a**) and chlorantraniliprole (**b**).

The predicted curves of residue dissipation are shown in Figure 5. The terminal residues of acetamiprid were 0.62 mg/kg by three-day intervals and 0.49 mg/kg by seven-day intervals. Although the residue exceeded the MRL after the third application when sprayed in three-day intervals, it was expected to decrease below the MRL seven days after the last spraying, which is the PHI of kimchi cabbage. Likewise, in the case of chlorantraniliprole, it was predicted that there was no possibility of exceeding the MRL in any case due to the relatively long PHI days (10 days). Based on these results, we concluded that an agricultural product safe from pesticide residue can be produced if the pesticides are sprayed following the directions for use on the pesticide label and are harvested after the PHI. This approach would also be helpful for predicting the residue amounts by different application intervals, numbers of treatment, and PHI.

## 3. Materials and Methods

### 3.1. Chemicals and Reagents

The pesticide reference standards of acetamiprid (purity: 99.9%) and chlorantraniliprole (purity: 99.8%) were purchased from Sigma-Aldrich (St Louis, MO, USA). HPLC grade acetonitrile and methanol were obtained from Fisher Scientific (Pittsburgh, PA). High purity formic acid (>99% LC-MS grade) was obtained from Sigma-Aldrich. Commercial QuEChERS extraction salts packets (4 g of magnesium sulfate, 1 g of sodium chloride, 1 g of trisodium citrate dihydrate, and 0.5 g of disodium hydrogen citrate sesquihydrate) and 2 mL tubes for dispersive solid-phase clean-up tubes containing 25 mg of primary secondary amine and 150 mg of magnesium sulfate were obtained from ULTRA Scientific (North Kinstown, RI, USA). Formulations of acetamiprid (suspension concentrate (SC), 6%) and chlorantraniliprole (Water dispersible granule (WG), 5%) for field experiments were purchased from a local agricultural market.

### 3.2. Pesticide Standard Solution

For the standard stock solutions, an accurate volume (25 mg) of the acetamiprid and chlorantraniliprole were weighed individually into a 25 mL volumetric flask and were dissolved by adding pure acetonitrile to achieve a concentration of 1000 μg/mL. Working solutions of 0.05, 0.1, 0.25, 0.5, 1.0 μg/mL were prepared from a stock solution by serial dilutions in acetonitrile. All stock and working standard solutions were stored in an amber glass vial at −20 °C until analysis.

### 3.3. Field Experiments and Pesticide Application

Kimchi cabbage was grown under open-field conditions at two different sites located in Icheon-si (Kyeonggi-do, Republic of Korea). The distance between the sites was about 16 km as a straight-line, and both sites were experienced farms that have been cultivating kimchi cabbage for over 10 years. During the field trial, average temperatures and relative humidities were 7.5 ± 2.5 °C and 48.6 ± 15.4%, respectively. The area of each field was about 80 m^2^, divided into three replicates and a control plot. Each plot was separated by 1 m^2^ of a buffer zone to avoid cross-contamination. For the spraying pesticide formulations, manufactured products of acetamiprid SC with 6% active ingredient (Dang Chan^®^, Kyung Nong Corporation, Seoul, Republic of Korea) and chlorantraniliprole WG with 5% active ingredient (Altacor^®^, Farm Hannong, Seoul, Republic of Korea) were diluted with water by a factor of 1:2000. The pesticides were applied once with a pressurized backpack sprayer (20 L) according to the manufacturer’s instructions. More than three heads of representative kimchi cabbage (>6 kg) were collected randomly from three replicated plots at 0 (2 h), 1, 2, 3, 5, 7, 10, and 14 days.

### 3.4. Sample Preparation and Extraction

All collected samples were placed into labeled polyethylene bags and transferred immediately to the laboratory. The kimchi cabbages that had merchantable quality were obtained after removing the outer leaves and root without any washing procedure, and their weight was measured. Because of the bulky volume of the kimchi cabbages, the samples were cut vertically and divided into four equal parts to minimize variation in the homogenization process. One of the quartered samples was homogenized with dry ice using a food processor (Hanil, HMF-3100S, Seoul, Republic of Korea). The powdered samples were put into a polypropylene bag and stored in a freezer at −20 °C until analysis.

The citrate-buffered QuEChERS method [31], which is the standard method used by the European Committee for Standardization (CEN), was used for the acetamiprid and chlorantraniliprole residue analysis. A 10 g sample of previously homogenized materials was weighed in a 50 mL polypropylene tube. Then, 10 mL of acetonitrile was added, and the tube was shaken vigorously using a mechanical shaker (1600 MiniG, SPEX Sample Prep, Metuchen, NJ, USA) for 1 min. These tubes were placed in an ice bath for about 10 min in advance. Then, the QuEChERS extraction package (4 g of magnesium sulfate, 1 g of sodium chloride, 0.5 g of disodium hydrogen citrate sesquihydrate, and 1 g of trisodium citrate dihydrate) was added and left to cool. After shaking again using the mechanical shaker for 1 min, the tubes were centrifuged at 3500 rpm for 5 min. An aliquot (1 mL) of the extract was transferred to the dispersive SPE tube, which contained 25 mg of PSA and 150 mg of magnesium sulfate, followed by vortexing for 30 s. After centrifuging at 13,000 rpm for 5 min, the clean upper layer (500 µL) was transferred into a 2 mL-amber vial, and 500 µL of acetonitrile was added prior to injecting into the UHPLC-MS/MS. For the matrix-matched standards, the blank extracts treated with the method described above were mixed at a ratio of 1:1 (*v*/*v*) with the solvent standard solutions of each concentration.

### 3.5. Instrumental Conditions

A UHPLC system (Nexera UHPLC, Shimadzu Corporation, Kyoto, Japan) equipped with an LCMS-8040 LC-MS/MS triple quadrupole system (Shimadzu Corporation, Kyoto, Japan) was used for quantification of target pesticides. The MS instrumental conditions involved in electrospray ionization (positive mode) were as follows: Capillary voltage of 4.0 kV, a heat block temperature of 400 °C, a desolvation line temperature of 250 °C, a nebulizing gas flow rate of 3.0 L/min (N_2_), and a drying gas flow rate of 15.0 L/min (N_2_). The chromatographic separation was achieved using a Phenomenex (Torrance, CA) Kinetex C18 column (100 × 2.1 mm, 2.6 μm). The column oven temperature was maintained at 40 °C, and the injection volume was 5 μL. The mobile phase consisted of water with 0.1% formic acid (A) and methanol with 0.1% formic acid (B) at a flow rate of 0.2 mL/min. To obtain desirable chromatographic peaks, two different gradient programs for each pesticide were used as follows (mobile phase B%): Acetamiprid—0 min, 10%; 1 min, 10%; 5 min, 95%; 7 min, 95%; 8 min, 100%; 9 min, 10%; 12 min, 10%, chlorantraniliprole—0 min, 5%; 0.5 min, 5%; 2 min, 95%; 5 min, 95%; 6 min, 5%; 7 min, 5%. To optimize the MRM transitions for each compound, the solvent standard solutions at 1 µg/mL were injected directly without the analytical column. After obtaining a full scan, the spectrum ranged between 50–500 *m/z*, and the [M + H]^+^ ions that gave the highest intensity were chosen as a precursor ion. Then, the product ions and the optimum collision energy were optimized by product ion scanning in the various CE voltages (0–50 eV).

### 3.6. Method Validation

The analytical method was validated through LOQ, i.e., linearity of the calibration curve, and recovery test. The LOQs were set to the lowest detectable concentration having a signal-to-noise ratio above 10. Matrix-matched calibration was employed to quantify the pesticide residues at six-points ranged from 0.025 to 1.0 µg/mL. The linearity was evaluated by the values of the correlation coefficient (*r*^2^) from the calibration curves. A recovery test was conducted to evaluate accuracy and precision at the different concentrations 10 and 50 times the LOQ levels (*n* = 3). Recoveries (%) were calculated as the percentage of agreement between the known spiked concentration of pesticides and the detected concentration of the recovery test. Accuracy and precision were evaluated by the mean recoveries and the RSD (%) within the replicates. During the period of sample storage in the freezer, the stability of the pesticide residue was evaluated. Blank samples spiked with acetamiprid, and chlorantraniliprole at a concentration of 0.25 mg/kg (*n* = 3) were stored in the same freezer (−20 °C in darkness) with the field samples as described above.

### 3.7. Statistical Analysis

The dissipation patterns of acetamiprid and cyantraniliprole in kimchi cabbage over time were expressed by the following first-order kinetics, which is a function of exponential decay. The biological half-lives, which means the time required for the initial residue to decrease by ½, were also calculated as follows [31,48].

*C_t_* = *C*_0_ × *e*^−kt^, DT_50_ = ln2/*k* (1)

where *C*_0_ is the initial residue concentration of pesticides from field experiments, *t* is the days after pesticide application, and *k* is the rate constant of dissipation.

According to the dissipation pattern data and MRL, the PHRLs on the day before harvest that are the allowable limit of the pesticide residues before harvest, were estimated from 15 to 0 days before harvest based on the following equation.

PHRL*_d_* = MRL × *e^kmin^*^× *d*^ (2)

Here, MRL is the maximum pesticide residue limits of each pesticide in kimchi cabbage (Republic of Korea), and *d* is the days remaining until harvest. The *k_min_* denotes the minimum rate constant of dissipation that refers to the lowest value in the confidence interval of the regression coefficient by the Student’s *t*-test at a 95% confidence level. All statistical calculations were calculated using Microsoft Office Excel.

## 4. Conclusions

We investigated the dissipation patterns of acetamiprid and chlorantraniliprole residues in an open-field system for kimchi cabbage cultivation. To determine the residues, simple and rapid analytical methods were developed and validated using a UHPLC-MS/MS. In the field study, the dissipation dynamics in the kimchi cabbages were discussed with biological half-lives of 5.2 and 6.3 days for acetamiprid and 10.0 and 15.2 days for chlorantraniliprole. Based on the dissipation equations, the PHRLs were suggested from 14 days to 1 day before harvest. The residue amounts predicted by different pesticide application intervals were estimated to be lower than MRLs because of the long PHI of the pesticide formulations. Overall, it is anticipated that these results both provide reliable data for understanding the fate of acetamiprid and chlorantraniliprole residues and useful guidelines to control the pesticide residues before harvest.

## Figures and Tables

**Table 1:** Multiple reaction monitoring (MRM) parameters and retention times for the ultra-high performance liquid chromatography coupled with tandem mass spectrometry (UHPLC-MS/MS) analysis.

Compound	Molecular Formula	t_R_^a^ (min)	Molecular Mass (g/mol)	Ionization	Precursor Ion > Product Ion (CE ^b^ Voltage)	
					Quantitation Transition	Qualification Transition
acetamiprid	C_10_H_11_ClN_4_	4.22	222.7	[M + H]^+^	222.9 < 126.1 (−20)	222.9 < 56.1 (−15)
chlorantraniliprole	C_18_H_14_BrCl_2_N_5_O_2_	3.65	483.1		483.8 < 452.9 (−19)	483.8 < 285.8 (−16)

^a^ t_R_: Retention time, ^b^ CE: Collision energy.

**Table 2:** Recovery results, linear regression parameters of calibration curve, limit of quantification (LOQ), and storage test in kimchi cabbage.

Compound	Spiked Levels (mg/kg)	Average Recovery (%) ± RSD ^a^ (%)	Calibration Curve			Linear Range (mg/kg)	MLOQ ^b^ (mg/kg)
			Slope	Intercept	r^2^		
acetamiprid	0.05	103.6 ± 4.3	13630.7			0.01–0.5	0.01
	0.25	113.9 ± 1.8		40959.5	0.9998		
	0.25 (Storage test)	114.1 ± 0.2					
chlorantraniliprole	0.05	80.8 ± 2.8	12233.4			0.01–0.5	0.01
	0.25	91.2 ± 0.7		50737.0	0.9964		
	0.25 (Storage test)	96.7 ± 5.5					

^a^ RSD: Relative standard deviation, ^b^ MLOQ: Method limit of quantification.

## Supplementary Materials

The following are available online, Figure S1: Average weight of the kimchi cabbages which were measured in each sampling day (*n* = 3), Figure S2: Temperature and humidity of the field (Icheon) during the experimental period
