# Supplementary material for: Dissipation Kinetics and the Pre-Harvest Residue Limits of Acetamiprid and Chlorantraniliprole in Kimchi Cabbage Using Ultra-Performance Liquid Chromatography-Tandem Mass Spectrometry
Source: Molecules. 2019 Jul 18;24(14):2616. doi: 10.3390/molecules24142616 (PMC6680825; doi:10.3390/molecules24142616)
Supplement: Supplementary file 1 [file molecules-24-02616-s001.pdf]

molecules

Supplemental data

Dissipation Kinetics and the Pre-Harvest Residue Limits of Acetamiprid and Chlorantraniliprole in Kimchi Cabbage using Ultra-Performance Liquid Chromatography-Tandem Mass Spectrometry

Jonghwa Lee [1,2,†], Byung Joon Kim [1,†], Eunhye Kim [1] and Jeong-Han Kim [1,*]

[1] Department of Agricultural Biotechnology and Research Institute of Agriculture and Life Sciences, Seoul National University, Seoul, 08826, Republic of Korea; payton20@snu.ac.kr (B. K.); cocopam777@snu.ac.kr (E. K.)
[2] Department of Veterinary and Animal Sciences, University of Massachusetts, Amherst, MA 01003, United States; jhlee006@gmail.com (J. L.)
* These authors contributed equally to this paper as first author.
* Correspondence: kjh2404@snu.ac.kr; Tel.: +82+2-880-4644

Received: date; Accepted: date; Published: date

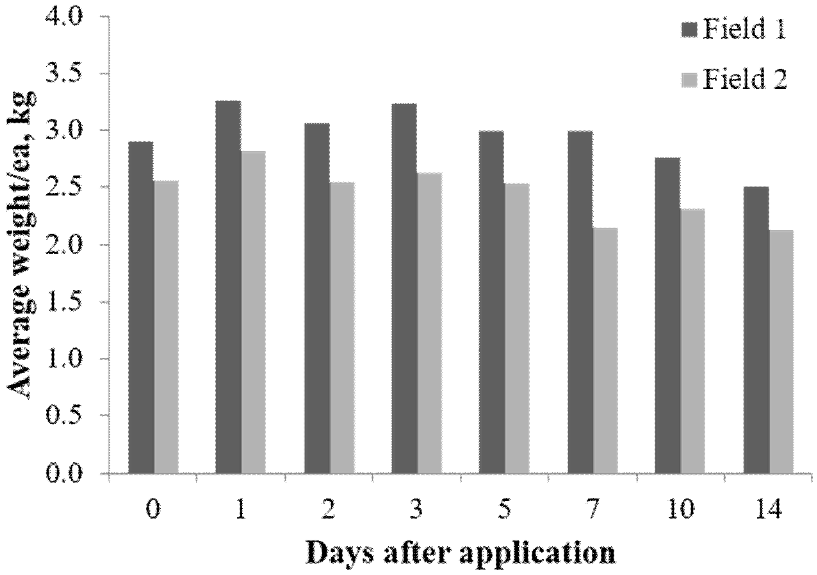

Figure S1. Average weight of the kimchi cabbages which were measured in each sampling day (*n*=3).

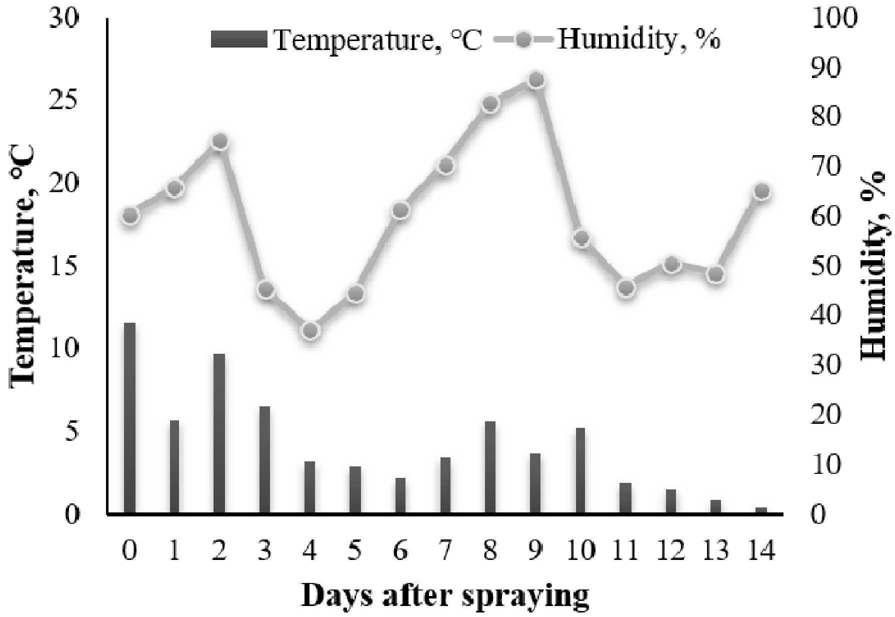

Figure S2. Temperature and humidity of the field (Icheon) during the experimental period
